# Decoding Genetic Markers of Multiple Phenotypic Layers Through Biologically Constrained Genome-To-Phenome Bayesian Sparse Regression

**DOI:** 10.3389/fmmed.2022.830956

**Published:** 2022-03-30

**Authors:** Marie Deprez, Julien Moreira, Maxime Sermesant, Marco Lorenzi

**Affiliations:** ^1^ University of Côte d’Azur, Nice, France; ^2^ INRIA, Epione Project-Team, Valbonne, France

**Keywords:** genome, phenome, biological constraint, bayesian, variational dropout, sparse regression

## Abstract

The applicability of multivariate approaches for the joint analysis of genomics and phenomics information is currently limited by the lack of scalability, and by the difficulty of interpreting the related findings from a biological perspective. To tackle these limitations, we present Bayesian Genome-to-Phenome Sparse Regression (G2PSR), a novel multivariate regression method based on sparse SNP-gene constraints. The statistical framework of G2PSR is based on a Bayesian neural network, were constraints on SNPs-genes associations are integrated by incorporating *a priori* knowledge linking variants to their respective genes, to then reconstruct the phenotypic data in the output layer. Interpretability is promoted by inducing sparsity on the genes through variational dropout, allowing to estimate the uncertainty associated with each gene, and related SNPs, in the reconstruction task. Ultimately, G2PSR is conceived to prevent multiple testing correction and to assess the combined effect of SNPs, thus increasing the statistical power in detecting genome-to-phenome associations. The effectiveness of G2PSR was demonstrated on synthetic and real data, with respect to state-of-the-art methods based on group-wise sparsity constraints. The application on real data consisted in an imaging-genetics analysis on the Alzheimer’s Disease Neuroimaging Initiative data, relating SNPs from more than 3,500 genes to clinical and multi-variate brain volumetric information. The experimental results show that our method can provide accurate selection of relevant genes in dataset with large SNPs-to-samples ratio, thus overcoming the main limitations of current genome-to-phenome association methods.

## 1 Introduction

Multi-omics data integration is an ever-growing field at the crossroad between biology and statistical learning. The goal of multi-omics analyses is to reveal novel insights on complex biological systems, through the combined analysis of multiple data types (Civelek and Lusis, 2014). Multi-omics data approaches are often designed to account for phenotypic information, providing quantitative features about an individual’s clinical or biological condition. Imaging-genetics is a typical application domain, in which genomic data under the form of Single-Nucleotide Polymorphisms (SNPs) are jointly analyzed with medical imaging information, composed of high-dimensional imaging quantitative traits (Shen and Thompson, 2020). The analysis of these complementary data types has proven quite successful to improve the discovery of genetic risk factors of complex and rare diseases, for example when applied to age-related macular degeneration, obesity, schizophrenia and Alzheimer’s Disease (Visscher et al., 2017). Nevertheless, current approaches to jointly analyze genomic and phenotypic information face multiple limitations due to genetic and imaging data’s inherent complexity and dimensionality.

The basic form of analysis between genomic and phenotypic information is genome-wide association studies (GWAS) (Tam et al., 2019), based on mass-univariate testing of associations between SNP and phenotypic features. Multiple comparison correction methods are generally used to mitigate the risk of false discovery, at the expense of potentially reduced detection power (Lindquist and Mejia, 2015). To compensate for the large data dimensionality, the number of association tests can be reduced by aggregating either the genomic or phenotypic features (Zhang et al., 2014). An intrinsic limitation of GWAS is due to the relatively small effect size of SNPs on the phenotypic features. When considered individually, most SNPs account for less than 1*%* of the variance in brain-imaging quantitative traits. This aspect motivates the development of strategies combining the effect of multiple SNPs to increase the detection power. These strategies take inspiration from known biological associations (Linkage Disequilibrium blocks) or structures (genes) to potentially reduce the dimensionality and improve the statistical power of genomic analysis (Hibar et al., 2011; Ge et al., 2012). To this end, more recent approaches have been proposed based on different modelling rationale and complexity, from multivariate regression (Vounou et al., 2012; Greenlaw et al., 2017) to deep learning methods (Wang et al., 2018).

For example, neural network architectures allow the identification of compressed representation of the data in the bottleneck layers, potentially allowing more accurate integration of the complex interaction between high-dimensional features (Schmidt et al., 1992). For instance, Wang et al. (2018) proposed an additive model via Feed-forward Neural networks with random weights to assess the role of each genetic feature independently in the prediction. Yet, neural network architectures require optimizing a large number of parameters, which thus negatively affects the reliability of these approaches in studies with low sample size and/or when the number of genetic features analyzed is large. In an attempt to mitigate this limitation, several methods have been proposed to induce sparsity in the model parameters and their associated features (Simon et al., 2013; Zhu et al., 2014; Greenlaw et al., 2017; Zhu et al., 2017). The study (Wang et al., 2012) presented a Group-Sparse Multi-task Regression and Feature Selection method structured as a sparse model based on *l*_2,1_ − norm regularization, to perform feature selection at both the group/gene-level and the SNP level. This kind of strategy improves the interpretability of the model results, as non-relevant SNPs and their associated genes can be readily pruned by the associated parameters. However, this kind of approach comes with an increased computational burden, limiting the number of genes and SNPs that can be analyzed. Scaling to a large number of genes without pre-selection remains a challenge in Genome-to-Phenome association studies, and most of the current applications have been demonstrated on dataset composed of up to few hundred of genes (Wang et al., 2012; Zhu et al., 2014; Greenlaw et al., 2017; Wang et al., 2018).

All in all, the applicability of current multivariate genome-to-phenome modeling approaches is limited by the lack of scalability, and by the difficulty of interpreting the related findings from a biological perspective. To tackle these limitations, in this paper we present Bayesian Genome-to-Phenome Sparse Regression (G2PSR), a novel multivariate regression method based on sparse SNP-gene constraint. G2PSR is conceived to alleviate the need for multiple testing correction and considers SNPs combined effect, thus increasing the analysis detection power. Our approach is orthogonal to other approaches to sparse neural networks (Lemhadri et al., 2021). The statistical framework of G2PSR is based on a Bayesian neural network, were constraints on SNPs-gene associations are integrated by encoding our knowledge on SNPs mapped in the same transcribed region, exons and introns, and promoter region to reduce genomic data dimensionality. This constraint maps the input SNPs into the corresponding genes represented in the intermediate layer of the network. We induce sparsity on the genes through variational dropout, to estimate the uncertainty associated with each gene (and related SNPs) in reconstructing the phenotypic features (output layer). The effectiveness of G2PSR is here demonstrated on synthetic and real data, with respect to state-of-the-art methods based on group-wise sparsity constraints. The real data application consisted of an imaging-genetics analysis on the Alzheimer’s Disease Neuroimaging Initiative (ADNI) data, relating SNPs from more than 3,500 genes to clinical scores and multivariate brain volumetric information. The experimental results show that our method can provide accurate selection of genes-of-interest in a dataset with a large SNPs-to-samples ratio, thus overcoming the main limitations of current genome-to-phenome association methods.

## 2 Materials and Equipment

In this section we describe synthetic and real data used to develop, optimize and test our Bayesian Genome-to-Phenome Sparse Regression (G2PSR) framework presented in the Methods section. We analyzed genomic data, such as genetic variations under the form of single nucleotide polymorphisms (SNPs), and phenotypic data, such as brain volume measurements or clinical examination scores. To test and develop our proposed method, we first generated synthetic data mimicking real genomic and phenotypic data with controlled genome-to-phenome associations (section 2.1). We then applied our method in a imaging-genetic case study in Alzheimer’s Disease, using data obtained from the Alzheimer’s Disease Neuroimaging Initiative (ADNI) (section 2.2).

### 2.1 Synthetic Data

Synthetic datasets were generated to mimic the properties of genomic and phenotypic data observed in real cases, with controlled genome-to-phenome associations. To evaluate G2PSR and benchmark it against state-of-the-art methods, we implemented a simulation system to generate pseudo-genomic data and associated phenotypic features. The pseudo-genomic data was generated to reproduce SNP information represented by multivariate arrays with entries 0, 1, or 2 corresponding to the number of alternative alleles found at the SNP location. The phenotypic data was subsequently defined to represent any discrete or continuous biological metric describing a phenotype, e.g., cognitive test results and volume measurements of different brain areas.

We denote by *N* the total number of SNPs, by *G* the total number of genes, by *T* the number of phenotype features, and by *S* the total number of samples to be generated. We define the *S* × *N* genotype matrix ***X***, where each row is partitioned in segments $\left(X_{g}\right)$ representing *g* = 1, … , *G* genes. The element *X*_*g*,*i*_ represents the SNP *i* mapped to the gene *g* generated to match the expected allele frequency observed in real data: *X*_*g*,*i*_ ∼ *MD*(*p*_0_), where *MD* is a multinomial distribution parameterized by $p_{0}=\left(p\left(x=0\right),p\left(x=1\right),p\left(x=2\right)\right)$ . We estimated this distribution using $∼390,\times ,10^{6}$ SNP values from the ADNI genetic dataset.

The *S* × *T* phenotype matrix ***Y*** is composed by *T* phenotypic features, obtained by concatenating genome-associated phenotype ***Y***_***G***_, and uncorrelated phenotype ***Y′***: ***Y*** = [***Y***_***g***_ ***,Y′***]. We define the genome-to-phenome association through a linear transformation, ***Y***_***g***_ = ***XV***, where each column *j* of the association matrix ***V*** has non-zero entries at the positions of the *n*_*g*_ SNPs associated to the gene *g* related to the phenotype *j*. The non-zero entries are randomly sampled from a Gaussian distribution N(0,Id) , while the uncorrelated phenotype features are random $Y^{\prime }∼N\left(\mu_{Y^{\prime }},\Sigma_{Y^{\prime }}\right)$ . The output phenotype is finally obtained as ***Y′′*** = ***Y*** + *snr* ^−1/2^ ⋅ ***ϵ***, where ***ϵ*** is standard Gaussian noise modulated by the desired signal-to-noise ratio *snr*. To determine the number of SNPs associated to each generated gene we estimated the distribution of the number of SNPs per gene in real genomic data. We used the number of SNPs in the 23′952 genes found in the ADNI genetic dataset.

### 2.2 ADNI Dataset

Data used in the preparation of this article were obtained from the Alzheimer’s Disease Neuroimaging Initiative (ADNI) database (adni.loni.usc.edu). The ADNI was launched in 2003 as a public-private partnership led by Principal Investigator Michael W. Weiner, MD. The primary goal of ADNI has been to test whether serial magnetic resonance imaging (MRI), positron emission tomography (PET), other biological markers, and clinical and neuropsychological assessment can be combined to measure the progression of mild cognitive impairment (MCI) and early Alzheimer’s disease (AD). Up-to-date information is available at www.adni-info.org. We selected clinical, genotypic and phenotypic data available in the ADNI-1/GO/2 datasets for 808 subjects.

The genetic data was filtered and processed chromosome by chromosome using multiple tools [vcftools (Danecek et al., 2011), bedtools (Quinlan and Hall, 2010) and PLINK 2.1 (Purcell et al., 2007; Shaun, 2014)]. We only selected Single Nucleotide Polymorphisms annotated in dbSNP (the NCBI database of genetic variation). We mapped them to exonic gene regions using *Homo sapiens* GFF annotation files (version GRCh.37, used by ADNI to map genetic variants). Variants were then filtered by their Minor Allele Frequency (MAF > 0.05). Missing data in the VCF files were imputed using the Sanger Imputation Server followed by post-imputation quality controls (imputation quality score, MAF and Hardy-Weinberg equilibrium). Lastly, SNPs with strong linkage disequilibrium (LD > 0.8) were filtered out, and all chromosomes were merged in a single file of annotated SNPs per sample. We obtained a genetic matrix of 485′101 SNPs grouped into 23′938 genes for 808 patients/samples. For our use case, we selected genes associated to the KEGG pathways (composed of 180 pathways including one focused on Alzheimer’s Disease). We obtained a genomic data matrix composed of 104′854 SNPs grouped into 3,953 genes.

The phenotypic data includes both clinical and brain volumes measurements. The clinical data is composed of six continuous variables generally recorded in memory clinics: the Alzheimer’s Disease Assessment Scale (ADAS) Cognitive Subscale (COG), Clinical Dementia Rating (CDR), the Mini-Mental State Examination (MMSE), the Functional Activities Questionnaire (FAQ) and the Rey Auditory Verbal Learning Test (RAVL immediate and forgetting). Imaging data were processed from structural MRI (grey matter only) to provide volume data on selected areas: Hippocampus and Entorhinal cortex. We therefore obtained a phenotypic matrix composed of 8 features matching the genetic data for 491 samples. Sample clinical status and demographics are described in Table 1.

**TABLE 1:** Summary of demographics and clinical information for the ADNI cohort analysed in our study.

Characteristics	Patients cognitively	Patients with mild	Patients with
	Normal (CN)	Cognitive impairment (MCI)	Dementia (D)
Sample size	150	200	141
Age—*yr*	73.07 ± 6.97	73.73 ± 7.74	74.83 ± 7.52
Male sex—no. (*%*)	67 (45*%*)	116 (58*%*)	81 (57*%*)
APOE − *ϵ*4 variant—no. (*%*)	34 (23*%*)	67 (34*%*)	87 (62*%*)

## 3 Methods

In this section, we describe the theoretical framework of G2PSR (Section 3.1). In Section 3.2, we present the experimental design used to optimize G2PSR, followed by the description of our benchmark experiments including the synthetic scenarios tested, the state-of-the-art methods used for comparison with G2PSR, and the performance metric used to evaluate their respective accuracies.

### 3.1 Bayesian Genome-To-Phenome Sparse Regression

**FIGURE 1:**
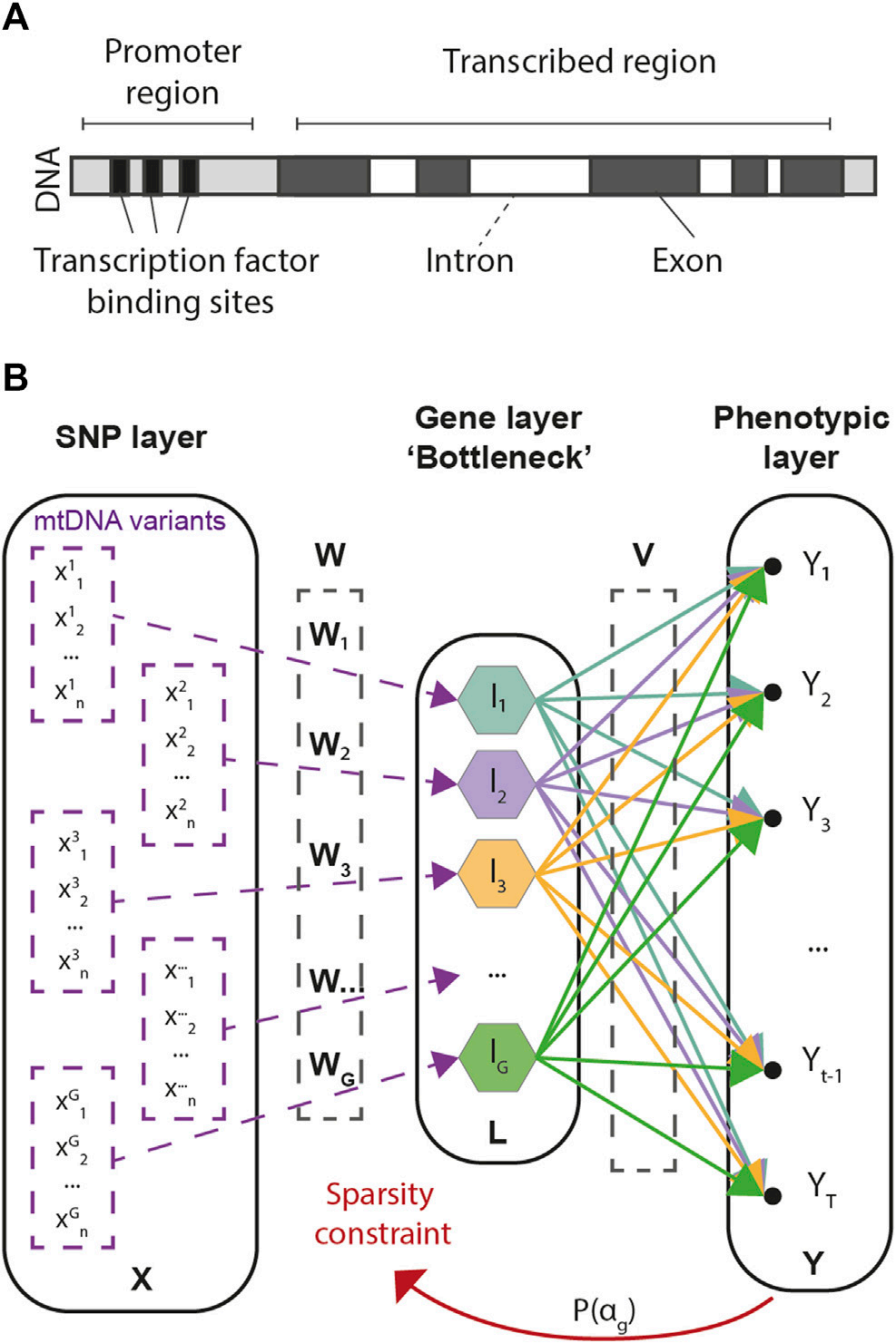
Genome-to-Phenome Sparse Regression architecture and description. **(A)** Gene structure describing the simplified constraint grouping SNPs into their associated genes, **(B)** G2PSR architecture from the input (SNP) layer to the constraint (Gene) layer and the output (Phenome) layer.

Our approach consists in a multivariate regression framework designed to predict multivariate phenotypic data from large arrays of SNP information. As the number of SNPs in GWAS is often an order of magnitude larger than the number of available samples, G2PSR is designed to account for biologically inspired constraints, in which known functional relationships across SNPs are accounted for under the form of group-wise sparsity penalization. The group-wise sparsity relationship across SNPs is designed to associate each SNP to its related gene, either within its transcribed region or in its regulation range (Figure 1A). This constraint defines the G2PSR network architecture from the input layer to the biologically constrained intermediate layer, that is finally transformed to reconstruct the output phenotypic layer (Figure 1B).

Let ***X*** the *S* × *N* input matrix representing the *N* SNPs studied for the *S* subjects. Each row of the matrix is partitioned in segments ***X*** ^***g***^, grouping the set of SNPs associated to the gene *g*. Let’s denote by *A* ^*g*^ the ensemble of SNPs indices associated to gene *g*, $X^{g}=\left\{x_{i},i\in A^{g}\right\}$ , *∀g* ∈ (1, … , *G*).

**FIGURE 2:**
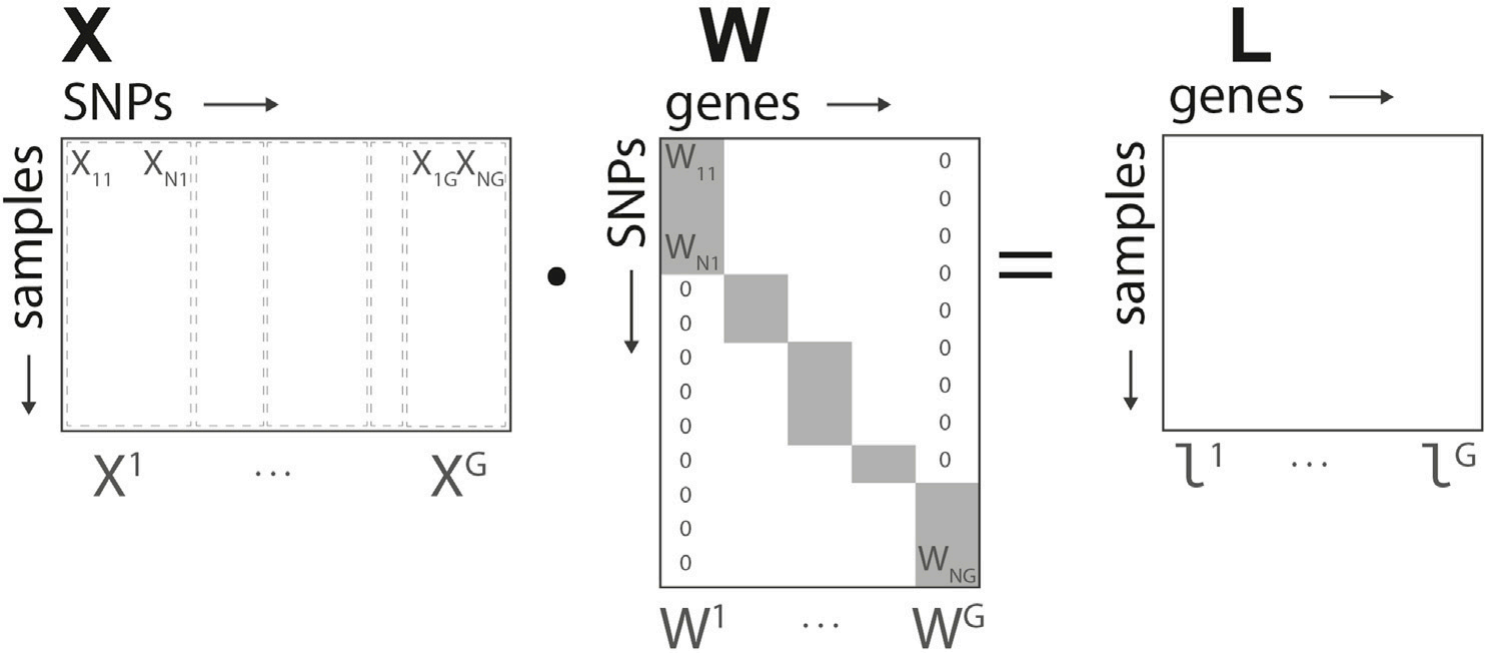
Genome-to-Phenome Sparse Regression architecture, detailed data structure for G2PSR generative model using the SNP-gene grouping constraint.

Based on the structure defined in Figure 2, for each gene we define a *N* × *G* linear transformation ***W*** mapping the input data to the gene representation, ***L*** = ***X*** ⋅ ***W***. The elements of the matrix ***W***_*i*,*g*_ are non-null if *i* ∈ *A* ^*g*^, and map the corresponding SNPs into the group-wise gene representation. In what follows, we denote by ***W*** ^*g*^ the non-null elements of each of the *G* columns of ***W***. The representation ***L*** = (***l*** ^**1**^, … , ***l*** ^***G***^) corresponds to the intermediate/gene layer of our network.

Finally, we assume that the phenotype is conditioned by the input layer through the likelihood:

$$Y∼N\left(L\cdot V,\Sigma \right),$$ (1)

where ***V*** is a *G* × *T* matrix allowing the reconstruction of the *T* phenotypic features from the gene layer ***L***, and Σ is the variance of the observational noise.

The parameters to be optimized in our model are the SNP-to-gene transformation ***W***, and the gene-to-phenotype transformation ***V*** or, in a more compact form, ***θ*** = {***W***, ***V***}. The solution of the inference problem can be obtained by maximizing the marginal likelihood *p*(***Y*** | ***X***) with respect to the distribution of ***θ***, which is usually an intractable problem. In this case, we can apply variational inference to learn a variational approximation *q*(***θ***) = {*q*(***W***), *q*(***V***)} to obtain an approximate posterior (Blei et al., 2017):

$$\begin{array}{cc} \log\left(Y|X\right) & =\log\int_{\theta }p\left(Y|\theta ,X\right)p\left(\theta \right)d\theta \\ & ⩾\underset{\text{A}}{\underset{︸}{E_{q\left(\theta \right)}\log\left(p\left(Y|\theta ,X\right)\right)}}-\underset{\text{B}}{\underset{︸}{KL\left(p\left(\theta \right)\|q\left(\theta \right)\right)}}. \end{array}$$ (2)

Equation (Bonda et al., 2009) represents the evidence lower bound (ELBO) associated to the inference problem (Blei et al., 2017). Accordingly, the optimization of the marginal likelihood can be solved by optimizing jointly the reconstruction term A with respect to the variational distribution *q*(***θ***), and the regularization term B represented by the Kullback Leibler divergence between the variational distribution and the prior *p*(***θ***). This problem can be efficiently solved by using standard optimization techniques based on backpropagation and reparameterization trick (Kingma and Welling, 2014).

To introduce biologically inspired constraints in the proposed G2PSR framework, the parameterization ***θ*** is specified in the following section.

#### 3.1.1 Regularization *via* Variational Dropout

To incorporate biological constraints in our framework, inspired by the seminal works (Hibar et al., 2011; Wang et al., 2012; Greenlaw et al., 2017; Lu et al., 2017), we impose group-wise penalization to the weights ***W*** ^***g***^ mapping the input SNPs to the common gene. The idea is that during optimization the model is forced to jointly discard all the SNPs mapping to genes which are not relevant to the predictive task, by setting to zero the associated parameters. To this aim, coherently with the variational approach detailed in section 3.1, we extend variational dropout as a group-wise regularization technique. Following (Molchanov et al., 2017), we parameterize the variational approximation *q*(***W*** ^***g***^) such that each element is defined as $W_{i}^{g}∼N\left(\mu_{i}^{g};\alpha_{g}.{\mu_{i}^{g}}^{2}\right)$ (Kingma and Welling, 2014), where the parameter *α*_*g*_ is optimized to quantify the common uncertainty associated with the ensemble of SNPs contributing to the gene *g*.

While the formulation is general, in what follows we restrict variational inference to the parameters **W** only, while optimizing the parameters **V** through maximum likelihood.

#### 3.1.2 Variational Dropout and Sparsity

According to Molchanov et al. (2017), the corresponding Kullback-Leibler divergence compatible with the proposed variational parameterization can be approximated by:

$$D_{KL}\left(q\left(W_{i}^{g}\right),q\left(W_{i}^{g}\right)\right)\approx -k_{1}\sigma \left(k_{2}+k_{3}\ln\left(\alpha_{g}\right)\right)+0.5\ln\left(1+\alpha_{g}^{-1}\right)+k_{1},$$ (3)

where *k*_1_ = 0.635 76, *k*_2_ = 1.873 20, *k*_3_ = 1.486 95 and *σ*(.) is the sigmoid function.

During the optimization of *D*_*KL*_ the sparsity arises naturally as it keeps *α*_*g*_ large for the less relevant features associated to larger uncertainty, while minimizing its value for the most relevant ones. Therefore, *α*_*g*_ is inversely proportional to the relevance of the gene in reconstructing the phenotypic features.

### 3.2 Synthetic Experiments

We used synthetic genome-to-phenome datasets to study the behaviour and evaluate the accuracy of G2PSR. We designed a “reference” dataset with fixed parameters described in Table 2, and produced multiple testing scenarios based on variants of this reference scenario, obtained by modifying a single parameter of interest while keeping the others fixed, Table 3. Each scenario was generated with ten replicates to assess the variability of the results.

**TABLE 2:** Synthetic dataset fixed attributes used to generate ‘reference’ scenario.

Attribute description	Value
Number of genes	200
Number of phenotypic features	15
Number of relevant genes	5
Number of samples	500
Noise level	20

**TABLE 3:** Dataset attributes, varied one-at-a-time in the prescribed ranges, and used to generate scenarios according to section 2.1.

Attribute description	Fixed value	Iteration list
Number of genes	200	20 50 100 200 500 1000 2000 5000 10 000
Number of phenotypic features	15	1 2 5 10 15 20 30
Number of relevant phenotypic features	15	4 15 as numbers OR 20, 50, 100% as percentage
Number of relevant genes	5	1 2 5 10 15 20
Number of samples	500	20 50 100 200 500 1000 2000
Noise level	20	0 20 50 80 100 150 200 300

#### 3.2.1 G2PSR Optimization

As described in the previous section, G2PSR is a Bayesian neural network using a biologically inspired constraint to reduce the number of genomic features to consider compared to the dataset sample size. However, in most cases, genomic data’s dimensionality still far exceeds the number of samples available and hence the number of associated parameters to estimate in the model. According to Li et al. (2020), G2PSR can be considered an over-parametrized regime and has thus the capacity to potentially (over-)fit any set of phenotypic features. To prevent such behaviour of the model, we define an optimal strategy to identify the number of iterations needed by the model to achieve the maximum performance while preventing overfit. This strategy is based on the control of key optimization parameters, such as training loss and estimated noise variance, to identify an early stopping strategy and optimize its performance.

**FIGURE 3:**
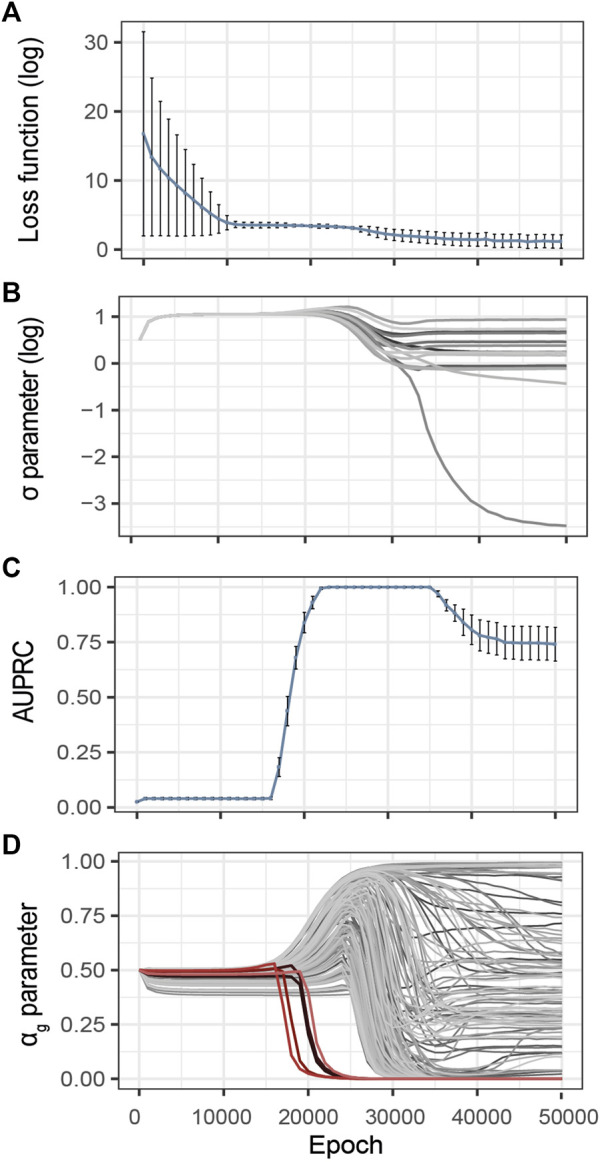
Evolution of the G2PSR parameters during the optimization process (Epoch/Iterations). **(A)** Loss value, **(B)** *σ* parameter, estimated noise variance, used to reconstruct the output (***Y***), each line correspond to a phenotypic feature, **(C)** *α*_*g*_ parameter per gene (sparsity metric), red lines correspond to relevant genes, grey lines correspond to non-relevant genes, **(D)** Evolution of the Area under the Precision-Recall Curve (AUPRC) estimated at the gene level using the *α*_*g*_ parameter. Error-bars show the standard deviation at each measured epoch (10 replicates).

Inspired by the heuristics described by Li et al. (2020), we control the evolution of the training loss and estimated noise variance during the optimization process (Figures 3A,B). The idea is that over-optimization of the loss is characterized by a consequent decrease of the estimated variance of the observational noise *σ*, thus pointing to over-fitting. These two metrics are thus complementary to identify model over-fitting.

To illustrate this strategy, Figure 3 provides an example of training on synthetically generated data. We applied G2PSR on the synthetic reference dataset described earlier (Table 2). Additionally to the loss and reconstruction error (Figures 3A,B), we also studied our model’s accuracy to identify the phenotype-relevant genes (Area Under the Precision-Recall Curve, AUPRC) and the corresponding *α*_*g*_ parameters which serve to quantify the genes uncertainty to reconstruct the phenotypic features (Figures 3C,D). From this example we observe that, after 25′000 epochs, over-fitting appears under the form of decrease in both training loss and noise variance. We observed that at 23′000 epochs G2PSR reaches its top accuracy, which later decreases when further optimizing beyond 35′000 epochs. From the analysis of the *α*_*g*_ parameters we determine that, before the decrease of the loss and *σ* values (∼ 20′000 epochs), the model correctly identifies the relevant features (i.e. *p*(*α*_*g*_) of significant genes) essentially ignoring the noisy ones (Figure 3D).

#### 3.2.2 Benchmark Experiments

We evaluated the performance and accuracy of G2PSR when compared to two state-of-the-art methods: Sparse Group Lasso (SGL) (Simon et al., 2013), and Bayesian Group Sparse Multi-Task Regression (BGSMTR) (Greenlaw et al., 2017) (Wang et al., 2012). We selected these methods to obtain a comparison with respectively a general sparse regression approach (SGL), and with a specifically designed method to relate genetic information with phenotypic data using biologically inspired constraints (BGSMTR). Similarly to G2PSR, we optimized the parameter settings of each method to retrieve the best performance in each tested scenario.

SGL is an extension of the group Lasso regulariser inducing parameter-wise sparsity (sparsity both at the covariates and at the group level). In our setting we used the SNPs as covariates and their related genes as a group associated with the phenotypic data of interest. As SGL can only relate the genomic data to a single phenotypic feature at the time, we used the average accuracy metric obtained across all phenotypic features tested. We optimized SGL using cross-validation with different covariates and group-wise regularization/sparsity terms (*l*_1_ and *l*_2_ norms), using the optimal accuracy metric among all tested parameter combinations.

BGSMTR was designed to produce confidence estimates on the regression parameters in addition to the *l*_2,1_ − norm penalty at both SNP and gene-level. Using a multivariate prior based on Gaussian scale mixture, the method is based on Markov Chain Monte Carlo (MCMC) to obtain the posterior distribution and interval estimates. To select potentially significant SNPs, the authors suggest evaluating the 95*%* equal-tail credible interval for each regression coefficient and selecting those SNPs and their related genes where at least one of the associated credible interval excludes 0. BGSMTR can relate multiple phenotypic features and produce a regression coefficient for each SNPs along with confidence intervals. As the optimization of so many parameters is costly in computation time and resources, we only tested BGSMTR with all phenotypic features being associated with the genome. We optimized BGSMTR using the cross-validation method proposed by the authors.

For each testing scenario, we set a limit of 72 h of computation time per method, including the parameters optimization (SGL and BGSMTR *l*_1,2_ − norms). To obtain the best performance from both benchmarking methods, we perform the joint optimization of regression weights and sparsity parameters of SGL and BGSMTR through grid-search (see Supplementary Table S1).

#### 3.2.3 Accuracy Metrics

We evaluated and compared the accuracy of each method in identifying the phenotype-relevant genes using multiple classification metrics. Considering that the number of SNPs and genes relevant to the phenotype is relatively small compared to all the SNPs evaluated, we relied mainly on the Area Under the Precision-Recall curve (AUPRC) and the F-measure. However, we used the F-measure as a reference accuracy metric for the BGSMTR method since only the binary classification of the relevant SNPs and their associated genes was available.

## 4 Results

This section describes the experimental results of our benchmark of G2PSR on extensive synthetic experiments, and on the real data from ADNI.

### 4.1 G2PSR Benchmark

We evaluated the computational complexity of G2PSR against an increasing number of genes and SNPs. We then assessed the ability of G2PSR to associate genomic data with multiple phenotypic features, and finally we compared its accuracy in multiple synthetic experiments (Section 3.2).

#### 4.1.1 G2PSR Computational Complexity

**FIGURE 4:**
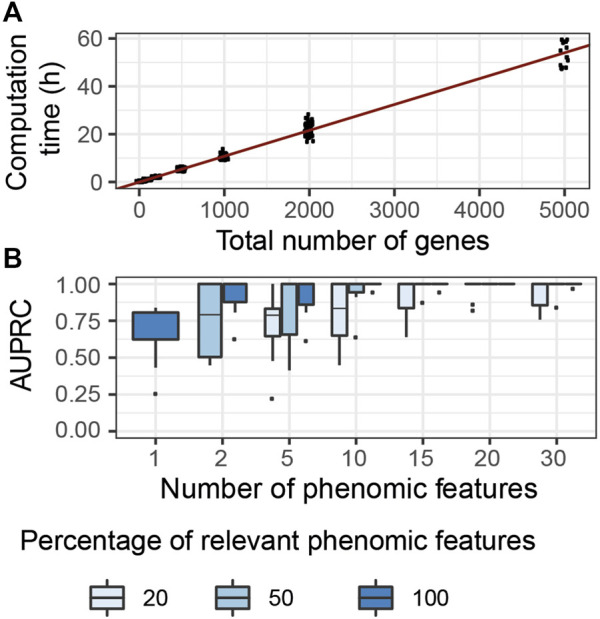
G2PSR model performance in specifically designed scenarios. **(A)** Computational time of G2PSR with respect to the total number of genes analysed. **(B)** Area Under the Precision-Recall Curve of our G2PSR model depending on the number of phenotypic features analysed and the percentage of genome-related phenotypic features among all features analysed (***Y*** = [***Y***_***g***_, ***Y′***], see section 2.1).

We evaluated the computational complexity of G2PSR by measuring the computation time required to run each tested scenario using a GPU node (DELL T630 GPU node, GeForce GTX 1080 Ti GPU), while using 50′000 number of epochs. We observed the most significant variation in computation time with respect to the total number of processed genes (Figure 4A), compared to other tested parameters (Supplementary Table S2). We noted a linear relationship between the computation time required and the total number of genes (and SNPs) analyzed. We estimated that, on average, the analysis of a thousand genes ( $∼20^{\prime }000$ SNPs) takes 12 h using our GPU.

#### 4.1.2 Genome-To-Multiple-Phenotypic-Features Accuracies

We evaluated the performance of our algorithm to associate genotypic data with genetically-relevant and genetically-independent phenotypic features in mixed proportions respectively (*Y*_*g*_ and *Y*′ in section 2.1), see Figure 4B. We noted that with a single phenotypic feature, G2PSR reaches a mean AUPRC of 0.60. When increasing the number of phenotypic features studied, the AUPRC improves even in the most challenging case with only 20*%* genetically relevant target features. This result can be explained by the redundancy induced by the relevant phenotypic features, wich allow to better identify causal genes. This result is encouraging, as most phenotypic features available for multi-omics data integration study are well-curated datasets with strong biological *a priori* in the selection of phenotypic features.

#### 4.1.3 Comparison With SGL and BGSMTR

**FIGURE 5:**
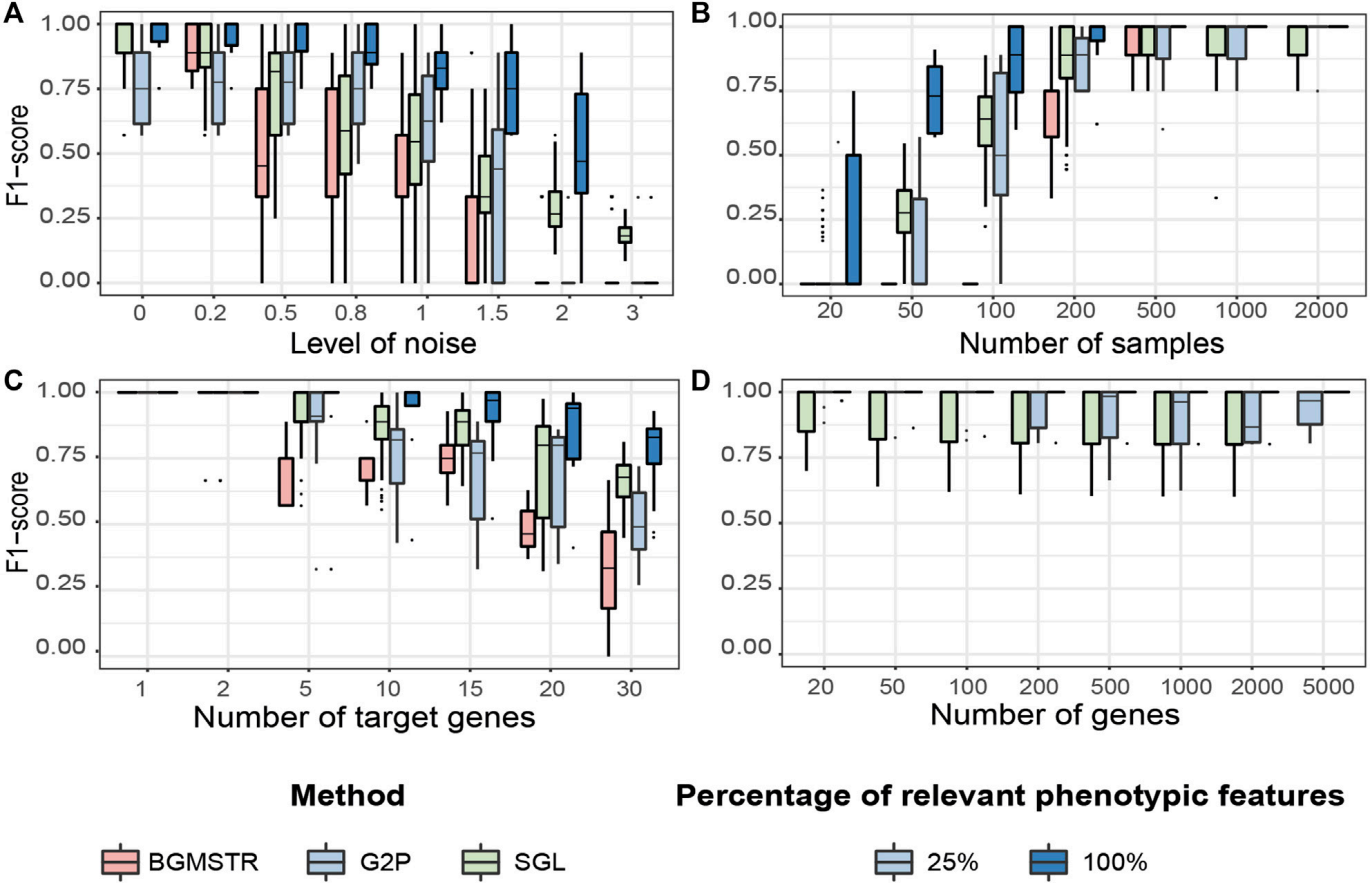
Testing benchmark of three group sparse methods applied to Genome-to-Phenome association according to varying generative parameters (Table 3). **(A)** Noise level in the phenotypic data. **(B)** Number of samples available in the data. **(C)** Number of “target”/relevant genes associated with the phenotype. **(D)** Total number of genes analysed (BGMSTR is not presented as its optimization in this scenario was computationally prohibitive). For our G2P model (blue boxplots), variations of the proposed scenarios are also shown with all 100*%* or only 25*%* of genome-related phenotypic features.

We compared the performance of three group-wise genome-to-phenome methods applied to synthetic scenarios (Tables 2, 3 and Figure 5). In the case of G2PSR, we evaluated its accuracy when considering 100*%* (G2PSR_100_) or 25*%* (G2PSR_25_) of gene-related phenotypic features, compared to SGL and BGSMTR, which were only tested by considering 100*%* relevant phenotypic features.

##### Noise Level

We observe that BGSMTR is the most sensitive method when faced with increasingly noisy phenotypic data, compared to SGL, which uses only one phenotypic feature and still maintained a better F-score (∼+0.15−0.25) , see Figure 5A and Supplementary Table S3. G2PSR_25_ has a lower performance than the other methods in low noise level scenarios (<0.5) but performs slightly better than SGL when the noise level is above 0.8 (∼0.15) . Overall, G2PSR_100_ out-performs all the compared methods in most tested scenarios (noise level <150% ).

##### Number of Samples

We observe that G2PSR_100_ out-performs all compared methods with generally less number of samples required (minimum ∼100 samples in the tested scenarios), see Figure 5B and Supplementary Table S4. SGL performs on average slightly better than G2PSR_25_ (∼0.05−0.20) with the same number of samples. BGSMTR remains the most sensitive method and required at least 500 samples to achieve similar efficiency than other comparison methods. We note that BGSMTR was still too costly to perform the optimization with an increasing number of samples ( >1000 samples).

##### Number of Target Genes

We evaluated the number of relevant genes correctly identified among all the genes tested. All methods have an optimal performance when the number of genes to identify is small (Figure 5C and Supplementary Table S5). However, as the number of significant genes increases, we observe a decrease in the overall performance across all tested methods. As previously observed, BGSMTR is the method for which performance decreases the most, while SGL still performs slightly better than G2PSR_25_ and G2PSR_100_, and is the method that maintains the highest performance ( >0.75% ). We hypothesize that most genome-to-phenome methods are well suited for analysing unbalanced data, with few relevant genes to identify.

##### Total Number of Genes

We observe that there is minimal variation in performance among all tested scenarios and also between G2PSR and SGL (BGSMTR was not evaluated here as it was too time-consuming to optimize), see Figure 5D and Supplementary Table S6. All methods have an average performance over 0.9 with a small decrease as the number of genes increases.

### 4.2 Real Data Application: Alzheimer’s Disease Use Case

We analyzed genomic, imaging and clinical data from 491 samples of the ADNI cohort. The analysed genomic data consisted of 104′854 SNPs grouped into 3′953 genes. Imaging and clinical data are used as phenotypic features, and are respectively composed of two-volume measurements (hippocampus and entorhinal cortex) and six cognitive scores (Materials section 2.2). To ensure that the optimization process did not lead to local minima, we applied G2PSR 10 times with 50′000 epochs to obtain average and standard deviation values for each parameter used in the analysis (loss value, *σ* and *α*_*g*_ parameters), see Supplementary Figure S1.

**FIGURE 6:**
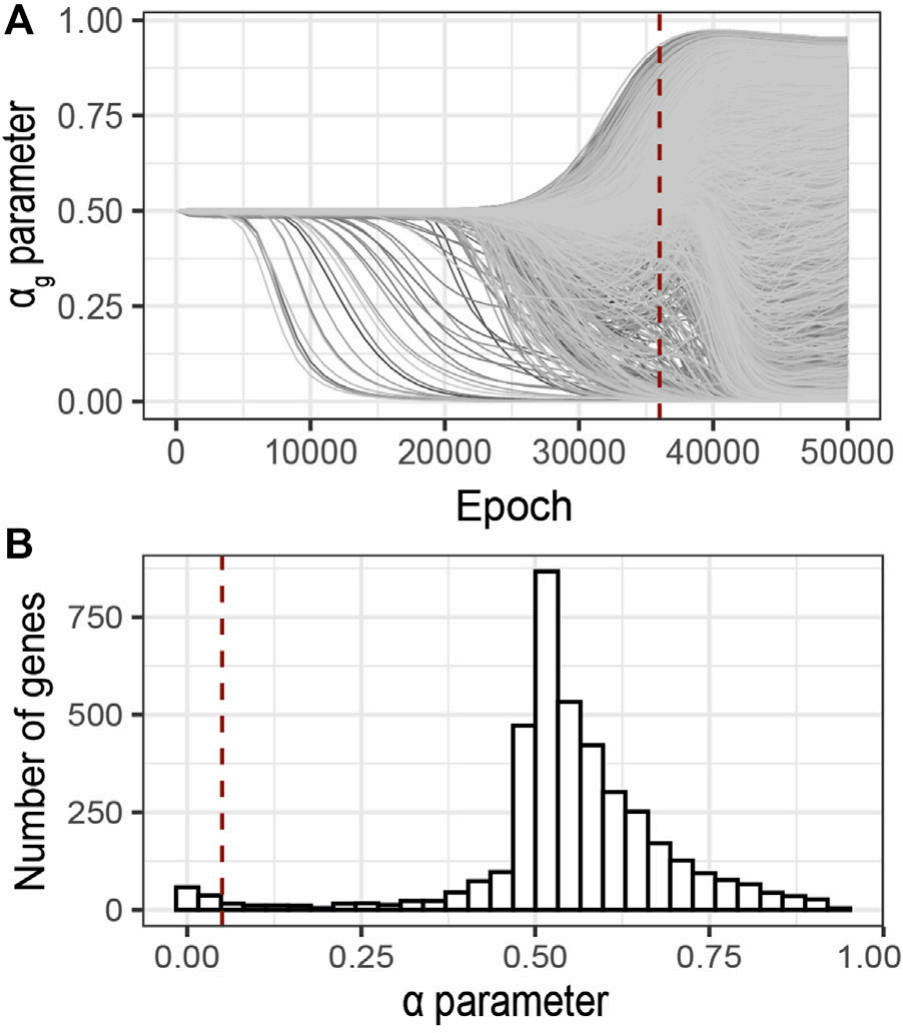
Identification of relevant genes using G2PSR on an ADNI dataset composed of 491 samples, 104854 SNPs grouped into 3,953 genes and 8 phenotypic features. **(A)** *α*_*g*_ parameter per gene through the optimization process, **(B)** Distribution of the *α*_*g*_ parameter at the optimal epoch, 36000 (dotted red lines in **A**). Red dotted line correspond to the nominal “relevance” threshold of 0.05 applied to the *α*_*g*_ parameter per genes.

**FIGURE 7:**
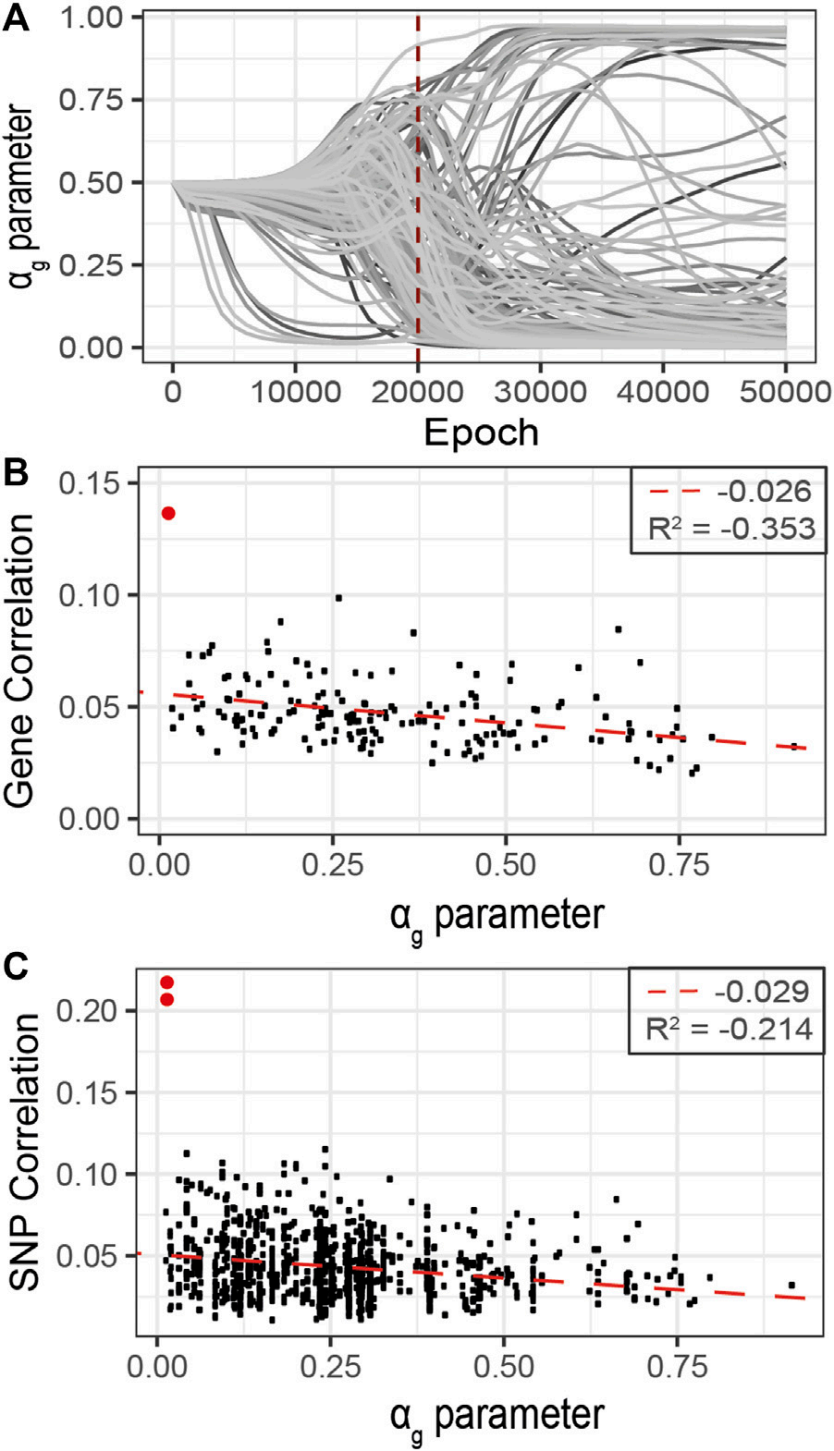
Refined analysis of relevant genes using G2PSR in the curated dataset identified in the primary analysis, 177 genes. **(A)** *α*_*g*_ parameter per gene through the optimization process, **(B)** Scatter plot of the gene-phenotypes correlation and the corresponding *α*_*g*_ value obtained with G2PSR (APOE gene in red), **(C)** Scatter plot of the SNP-phenotypes correlation and the corresponding *α*_*g*_ value obtained for their related genes (rs429358 and rs769449, APOE SNPs in red).

Following the early stopping strategy described above, we analyzed the sparsity parameters *α*_*g*_ at 36′000 epochs, Figure 6A. The distribution of the *α*_*g*_ values at the selected epoch shows a bimodal trend (Figure 6B). One mode of this distribution corresponds to minimal *α*_*g*_ values ( <0.05 , red dotted line), thus pointing to potentially relevant genes associated with the phenotype. From this analysis, we identified 177 genes with an *α*_*g*_ value lower than the nominal cut-off of 0.05 (Supplementary Table S7). Interestingly, the main known genetic risk-factor associated with AD, the gene APOE, was ranked 10th by *α*_*g*_ value in this experiment. This set of genes was used to filter the gene list and SNP data to perform a second optimization G2PSR round. Similarly to the previous analysis, we identified our early stopping strategy at 20′000 epochs and analyzed the corresponding sparsity *α*_*g*_ values of the 177 genes here considered (Figure 7A). We also performed a correlation analysis between each gene, their associated SNPs, and the ensemble of phenotypic features (Figures 7B,C and Supplementary Table S8). We observe a negative correlation (*R* ^2^ = 0.353) between the average gene-phenotype correlation and their respective *α*_*g*_ values obtained with G2PSR. In particular, top genes with a low *α* value have at least one associated SNPs with a notable correlation with the phenotypic features. The most relevant example is the APOE gene (red dots in Figures 7B,C), which is the top relevant gene and has two SNPs (rs429358, rs769449) with a correlation over 0.2 with phenotypic features (Table 4). Among the other top genes identified, some have not yet been well characterized as associated with the Alzheimer’s Disease but are described as involved in brain functions. For instance, the Methionine Adenosyltransferase 2B, MAT2B gene has been described as reducing ischemic brain injuries in rat (Li et al., 2019). The protein transporter THOC3, the NKD2-WNT signaling pathway inhibitor and the PI3-kinases, PIK3C2B, have been described in dysregulated pathways due to the APOE − *ϵ*4 toxicity in Human neurons (Najm et al., 2020; Li and De Muynck, 2021). We also identified genes such as PTPN11 (also known as protein tyrosine phosphatase SHP2), and MCM2, involved in cell maintenance and renewal, which have been characterized as risk factors for Alzheimer’s Disease (Bonda et al., 2009; Kim et al., 2019a).

**TABLE 4:** G2PSR results applied to the ADNI dataset. Top genes ranked by *α*_*g*_ parameter. Gene correlation with phenotypic features and their associated significative SNPs (with corresponding SNP correlation and Bonferroni corrected p-value).

Gene name	*α*_*g*_ value	Gene correlation	Relevant SNPs (correlation/p-value)
APOE	0.013 8	0.136 6	rs429358 (0.22/3.08*e* ^−08^)
			rs769449 (0.21/3.17*e* ^−07^)
MAT2B	0.019 9	0.049 0	
THOC3	0.021 0	0.040 6	
NKD2	0.033 1	0.045 4	
PTPN11	0.043 8	0.072 9	rs11614544 (0.08/0.94)
PIK3C2B	0.044 1	0.044 1	rs1008833 (0.11/0.34)
MCM2	0.051 2	0.054 0	rs11718485 (0.09/0.01)
GRK6	0.055 1	0.049 3	
RPL37	0.059 4	0.040 1	
EXOC3	0.062 0	0.038 6	

As the correlation per SNP with the phenotypic features rarely exceeds 0.1, this analysis suggests that the grouping constraint of G2PSR mapping SNPs into genes increases the detection power of our method to identify phenome-relevant genes.

## Discussion

This study proposes G2PSR, a Bayesian neural network accounting for biologically inspired constraints to provide Genome-to-Phenome sparse regression. Through the extensive benchmarking of our method on synthetic and real experimental scenarios, we demonstrate that G2PSR overcomes critical limitations of classical genome-to-phenome analyses. Through the SNPs-genes constraint, our method improves detection power, and reduces the number of parameters to optimize (thus minimizing the risk of overfitting) while simultaneously providing an interpretable neural network architecture through group-wise sparsity constraint based on a sound Bayesian framework.

Scalability is one of the main features of G2PSR, as most of published application cases have been currently limited to less than a thousand genes. For example, Zhu et al. (2014) proposed a Bayesian generalized low-rank regression (GLRR) applied to a dataset composed of 1′071 SNPs from 40 AD genes, while Lu et al. (2017). extended the above-mentioned GLRR model and applied it on the same dataset. In the study of Wang et al. (2012), the proposed model was applied to study 3′123 SNPs from 153 AD candidate genes. Our results show that despite the limited number of samples (similar to previous works), our method is able to analyse a far larger number of SNPs, while still providing relevant genome-to-phenome associations. In future work, we aim to test the scalability of G2PSR to whole genome analysis (full SNP dataset, 30 000 genes) so as to avoid completely the initial gene selection step, although we anticipate that larger sample size would be needed to reach adequate statistical power. We hypothesize that such improvements can be made through a multi-GPUs optimization or by using more efficient GPUs with a larger memory capability, as the number of parameters to account for increases linearly with the number of genes/SNPs studied.

By simultaneously integrating multiple phenotypic traits under a sparsity constraint, our application of G2PSR on ADNI demonstrates that our method can effectively process an extensive number of genes and SNPs and reliably identify relevant genes associated to the phenotype. We identified the well known risk-gene APOE (Liu et al., 2013; Sala Frigerio et al., 2019; Husain et al., 2021), with two SNPs highly correlated to the studied phenotypic features (rs429358 and rs769449). G2PSR also identified the following relevant genes: PTPN11 (Kim et al., 2019b), (regulation of the phosphorylation of the tau protein found to be in excess in AD cells), THOC3 (Najm et al., 2020) (protein transporter involved in the export of HSP mRNAs, necessary for the maintenance of brain cells), MCM2 (Bonda et al., 2009; Sala Frigerio et al., 2019) (involved in cell cycle aberrations in AD), GRK6 (Obrenovich et al., 2009; Guimarães et al., 2021) (which disturbed expression pattern has been identified in AD) and PIK3C2B (Liang et al., 2008; Liu et al., 2011; Maffei et al., 2018) (which dysfunction can cause hyperphosphorylation of the tau protein involved in AD onset). As we studied SNPs in the exonic region of each gene, we hypothesize that the variants might play a role in the gene function and/or expression, and produce an effect associated to the underlying pathological process. Nonetheless our method also identified genes for which the association with AD has not been demonstrated, such as MAT2B, NKD2 and RPL37. Through our complementary correlation analysis, we observe that each of these genes contain a SNP with a high correlation with the studied phenotype. These correlations correspond to the underlying signal picked up by G2PSR, and further analysis and validation are necessary to interpret the actual role of these genes in the pathophysiology of AD.

By construction, our approach does not optimize the direct identification of individual SNPs driving the genotype-phenotype association. G2PSR was rather designed to account for structural and functional constraint associating SNPs to their corresponding (mapped) genes thus increasing the detection power of phenotype-relevant genetic features while accounting for the dataset sample size. In our experiments, we illustrate how G2PSR can be used to robustly identify relevant genes, and we propose a two-step workflow to first screen among a large number of genes for relevant genome-to-phenome association with respect to the sample size, and then adopt a second level analysis with an increased statistical power (features-to-sample ratio) to pinpoint the most significant SNPs driving the gene-phenotype association. Our results demonstrate that G2PSR achieves an optimal selection of relevant genes associated with the phenotype, and thus can be completed with more targeted GWAS to better characterize the relevant SNPs among the selected genes. By comparison with the methods used to benchmark G2PSR, SGL and BGSMTR, which both allow direct identification of the phenotype-relevant SNPs, our method was develop to propose a compromise between directly interpretable results and the number of genes that can be evaluated simultaneously. As a consequence, we propose G2PSR as a screening tool to robustly identify relevant genes. The use of G2PSR ultimately circumvents the multiple comparison problem typical of standard GWAS applications by reducing significantly the number of genes and SNPs to be eventually evaluated in complementary “post-hoc” analysis.

Our analysis reveals several directions of improvement that could be implemented in future work. While reducing the number of genetic covariates, the gene constraint does not eliminate the correlation between SNPs close to each other on the genome through linkage disequilibrium (LD), and thus the correlation between their related genes. An improved group-wise constraint could account for the correlation between SNPs to produce relatively independent groups, for example by considering a mix of genes and LD blocks. Regarding the multi-omics analysis rationale, it would be relevant to introduce another data level into the G2PSR framework. For instance, gene expression data, such as from RNA-sequencing, could be used as an additional constraint in the neural network architecture, or as a prior on the weights in our variational scheme. Such an approach could improve the selection of genes relevant to the phenotype, potentially reduce the burden of the optimization process, and improve the interpretability of the model from a more mechanistic point of view.

To conclude, our study shows that G2PSR is an effective tool to identify genetic correlates of phenotypic features in the high-dimension/low-sample size regime, and can thus be employed in future challenging applications, such as imaging-genetics and rare disease analysis.

### Permission to Reuse and Copyright

Figures, tables, and images will be published under a Creative Commons CC-BY licence and permission must be obtained for use of copyrighted material from other sources (including re-published/adapted/modified/partial figures and images from the internet). It is the responsibility of the authors to acquire the licenses, to follow any citation instructions requested by third-party rights holders, and cover any supplementary charges.

## Data Availability

Publicly available datasets were analyzed in this study. This data can be found here: http://adni.loni.usc.edu/. Data publicly available at https://github.com/Inria-Asclepios/G2PSR.

## Supplementary Material

Supplementary Table S1 Average computation time per method in two tested scenarios, includes either oprimization of regression weights only or grid-search of sparsity parameters (*l*_1,2_ − norm for SGL and BGSMTR) and the corresponding optimization of regression weights.

Supplementary Table S2 Average computation time and standard deviation (in minutes) per tested scenarios. Each line corresponds to the varying attribute in the considered scenarios.

Supplementary Table S3 Benchmark results of three group sparse methods applied to Genome-to-Phenome association with varying noise level in the phenotypic data. Each line describes the method used, noise level tested, number of genome-related phenotypic features (15—100*%*, 4—25*%*), number of replicates and the mean F-score and standard deviation obtained.

Supplementary Table S4 Benchmark results of three group sparse methods applied to Genome-to-Phenome association with varying sample size. Each line describes the method used, sample size, number of genome-related phenotypic features (15—100*%*, 4—25*%*), number of replicates and the mean F-score and standard deviation obtained.

Supplementary Table S5 Benchmark results of three group sparse methods applied to Genome-to-Phenome association with varying significant genes. Each line describes the method used, number of target/significant genes, number of genome-related phenotypic features (15—100*%*, 4—25*%*), number of replicates and the mean F-score and standard deviation obtained.

Supplementary Table S6 Benchmark results of three group sparse methods applied to Genome-to-Phenome association with varying number of genes processed. Each line describes the method used, total number of genes in the dataset, number of genome-related phenotypic features (15—100*%*, 4—25*%*), number of replicates and the mean F-score and standard deviation obtained.

Supplementary Table S7 G2PSR results on the ADNI dataset. Table of the 3,953 genes analysed with their corresponding *α*_*g*_ value at the optimal number of epochs (36000).

Supplementary Table S8 G2PSR results on the refined ADNI dataset. Table of the 177 genes and their SNPs analysed. The table contains the genes *α*_*g*_ parameter at 20000 epochs, their average correlation with all phenotypic features and their corresponding SNPs correlation with each phenotypic feature and the associated correlated p-value.
