# Supplementary figures and images for: Decoding Genetic Markers of Multiple Phenotypic Layers Through Biologically Constrained Genome-To-Phenome Bayesian Sparse Regression
Source: Front Mol Med. 2022 Mar 30;2:830956. doi: 10.3389/fmmed.2022.830956 (PMC11285669; doi:10.3389/fmmed.2022.830956)

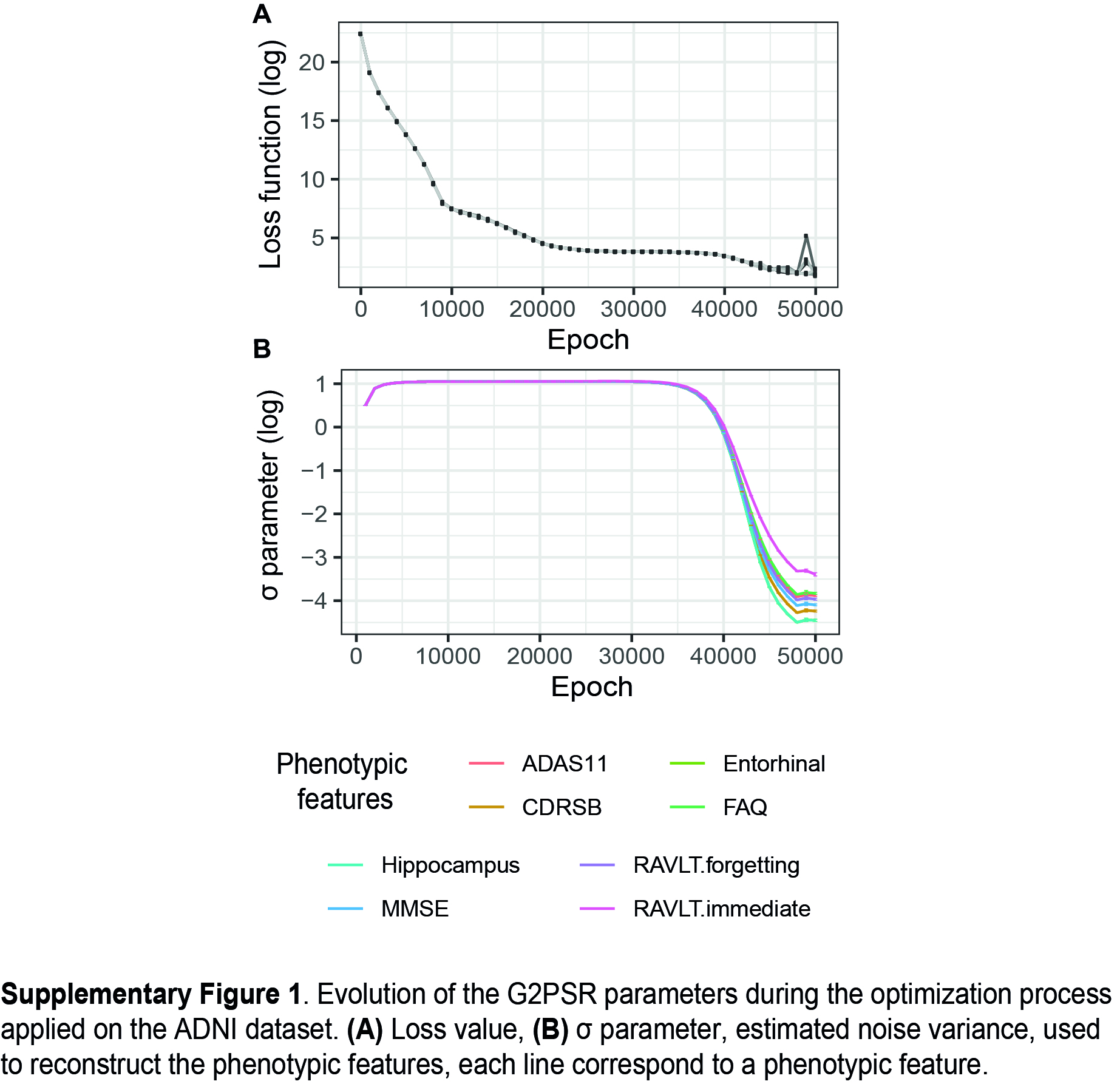

Supplement: Supplementary file 3 [file Image1.JPEG]
